# Limitations of using the DATASUS database as a primary source of data in surgical research: a scoping review

**DOI:** 10.1590/0100-6991e-20233545-en

**Published:** 2023-08-14

**Authors:** SOFIA WAGEMAKER VIANA, MATHEUS DANIEL FALEIRO, ANNA LUIZA FONTES MENDES, AMANDA CIPRIANO TORQUATO, CLARA PEREIRA OLIVEIRA TAVARES, BRENDA FERES, MIGUEL GODEIRO FERNANDEZ, ITALLO ROMERO MARQUES SOBREIRA, CAROLINE MARQUES DE AQUINO, SIMONE DE CAMPOS VIEIRA ABIB, FABIO BOTELHO

**Affiliations:** 1 - Kursk State Medical University - Kursk - Kurskaya Oblast - Rússia; 2 - Universidade Federal de Minas Gerais - Belo Horizonte - MG - Brasil; 3 - Universidade Vale do Rio Doce - Governador Valadares - MG - Brasil; 4 - Universidade Salvador - Salvador - BA - Brasil; 5 - Escola Bahiana de Medicina e Saúde Pública - Salvador - BA - Brasil; 6 - Centro Universitário Tocantinense Presidente Antônio Carlos - Araguaína - TO - Brasil; 7 - Universidade Nove de Julho - Sao Paulo - SP - Brasil; 8 - Universidade Federal de São Paulo, Departamento de Cirurgia Pediátrica - São Paulo - SP - Brasil; 9 - Hospital das Clínicas da Universidade Federal de Minas Gerais - Belo Horizonte - MG - Brasil; 10 - The Montreal Children’s Hospital, Harvey E. Beardmore Division of Pediatric Surgery - Montreal - Quebec - Canadá

**Keywords:** Epidemiology, Statistical Databases, Brazil, Epidemiologia, Bases de Dados Estatísticos, Brasil

## Abstract

**Objective:**

DATASUS is the Brazilian Public Unified Health System (SUS) department responsible for providing health data that are used as a primary source of data in several studies on surgery and surgical specialties although its main limitations have not been previously reviewed. The objective of this work is to synthesize information from studies on surgery that used DATASUS systems as a data source and to identify the main gaps in this platform.

**Methods:**

a scoping review was conducted according to the PRISMA-ScR method to identify papers on surgery, and other surgical specialties, that used the DATASUS platform as a primary data source. No restrictions were imposed regarding the type of study or year of publication. Grounded Theory was used to analyze the content of the articles.

**Results:**

248 works were initially analyzed and 47 were included in the final analysis of this study. The original articles included were published between 2009 and 2022 and the majority (12.76%, n=6) were published in the Journal of the Brazilian College of Surgeons. Retrospective studies (40.43%, n=19) were the most common type of study found. Content analysis of the articles identified four predominant domains in the scientific literature about the limitations of using DATASUS in surgical research: lack of data, reliability, precision and data integration.

**Conclusion:**

the information systems available in DATASUS are the largest source of information about the SUS, but the scientific literature on the quality of data available in these systems remains scarce and studies aimed at measuring this metric are necessary.

## RESUMO

**Objetivo:**

o DATASUS é o departamento do SUS responsável por disponibilizar dados de saúde que são empregados como fonte primária de dados em diversos estudos sobre cirurgia e especialidades cirúrgicas, embora principais limitações não tenham sido revisadas anteriormente. O objetivo deste trabalho é sintetizar as informações de estudos sobre cirurgia que utilizaram sistemas do DATASUS como fonte de dados, identificando as principais lacunas.

**Métodos:**

uma revisão de escopo foi conduzida de acordo com o método PRISMA-ScR para a identificação de trabalhos sobre cirurgia, e outras especialidade cirúrgicas, que utilizaram a plataforma DATASUS como fonte primária de dados. Nenhuma restrição foi imposta em relação ao tipo de estudo ou ano de publicação. A Teoria Fundamentada em Dados foi utilizada para a análise do conteúdo dos artigos.

**Resultados:**

248 trabalhos foram inicialmente analisados e 47 foram incluídos na análise final deste estudo. Os artigos originais incluídos foram publicados entre 2009 e 2022, maioria (12,76%, n=6) foi publicada na Revista do Colégio Brasileiro de Cirurgiões. Estudos retrospectivos (40,43%, n=19) foram o tipo de estudo mais comum encontrado. A análise dos artigos identificou quatro domínios predominantes na literatura científica acerca das limitações do uso de DATASUS em pesquisas em cirurgia: falta de dados, confiabilidade, precisão e integralização dos dados.

**Conclusão:**

os sistemas de informação dispostos no DATASUS constituem a maior fonte de informações sobre o SUS, porém a literatura científica sobre a qualidade dos dados dispostos nestes sistemas permanece escassa e trabalhos direcionados a mensurar essa métrica são necessários.

## INTRODUCTION

The Brazilian Public Unified Health System (SUS) has the Department of Information Technology (DATASUS), responsible for developing, researching, and incorporating information technologies that enable the implementation of systems in the health area^1^. It is also in charge of maintaining the systems and applications necessary to record, process, and make available health information originating from SUS’s health institutions, which are sent to the Health Assistance departments of the Ministry of Health. The main systems and databases that comprise the platform are the National Register of Health Establishments (CNES), the SUS Outpatient Information System (SIA-SUS), and the SUS Hospital Information System (SIH-SUS), whose function is to register all attendances resulting from hospital admissions financed by SUS by capturing data from Hospital Admission Authorization forms (AIHs)^1^ ^,^ ^2^.

SIH-SUS was originally developed as a financial system to handle payments for hospital services. Currently, it collects information on 60% to 70% of hospitalizations in the country in the Unified Health System^2^ ^,^ ^3^, not including hospitalizations in hospitals not linked to the SUS, whose expenses are directly charged to the user or paid by health insurance plans, which covers 28.5% of the Brazilian population^4^. In this system, hospitals individually submit a formal monthly report that includes data on a variety of diagnoses and procedure statistics^5^.

The Department of Informatics of the Unified Health System brings together the main Information Systems used for research with secondary data in Brazil. However, we should note that this is not the only source of existing health-related data, as other Information Technology centers are responsible for information systems with restricted access to requests and approvals by the responsible body6. Important examples within this definition include the Integrated Health Surveillance Platform (IVIS) and the Neonatal Screening Information System (SISNEO). However, in the surgical field, DATASUS still gathers the largest amount of information related to program control and federal fund transfers, making it possible to access information relevant to Brazilian demography, procedures, and their variables (number of procedures, mortality, hospital stay, cost of hospitalization and procedure, aspects related to geographic location, and establishment), and epidemiological profiles of hospitalizations^6^.

The purpose of making these health data available at the county level is to provide data capable of supporting objective analyzes of health, epidemiological situations, and financial resources. Thus, DATASUS provides, as primary sources, fundamental data for scientific research, including in the surgical area. Even so, the platform’s ability to provide data capable of supporting such analyzes has not been comprehensively reviewed in previous studies, with the aim of generating an intervention in the evaluations of the Brazilian health scenario. The objective of this scope review is to summarize the main limitations of the use of the DATASUS platform in studies on surgery, to serve as a basis for the construction of policies that will strengthen it.

## METHODS

Considering that this research is based on a review of the previously published scientific literature and that there was no direct involvement of patients, the analysis by an institutional ethics committee was waived.

### Study design

We conducted a scoping review on the limitations of using DATASUS as a primary source of data in works on surgery and other surgical specialties, in accordance with the guidelines of the Preferred Reporting Items for Systematic Reviews and Meta-Analyses method for scoping reviews (PRISMA-ScR).

We collected the data in four different databases, ending on March 27, 2023. From the studies included after the strategy, we applied the exclusion criteria and used Grounded Theory to analyze the content of the works included.

### Identification of studies

To identify relevant studies, we systematically searched PubMed, Web of Science, Embase, and Scopus platforms, using the terms [DATASUS] OR [SIM] OR [SIH] AND [cirurgia]. We included articles in Portuguese or English that cited at least one limitation of the use of the DATASUS platform in studies on surgery or other surgical specialties. Two authors (MDF, SWV) independently identified the studies out in two stages: (1) reading of the title and abstract; and (2) reading of the full text. Regarding the inclusion criteria adopted, the articles included in this study dealt with topics related to surgery, anesthesia, or obstetrics (SAO), as well as other surgical specialties, if they used the DATASUS platform as a primary source of data and if they commented on one or more limitations on the use of the platform. No restrictions were imposed regarding the type of study or year of publication. We excluded papers that did not address topics related to the research scope, that used another database as a data source, or that did not mention at least one DATASUS platform limitation.

**Figure 1.**
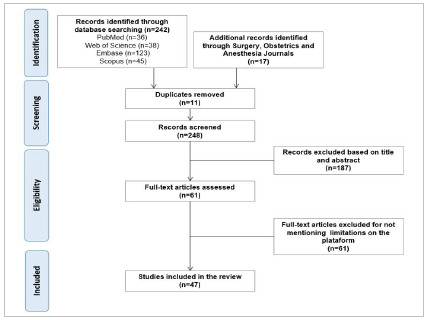

To increase the sensitivity of the search strategy, we conducted a complementary search for scientific papers in SAO journals and other surgical specialties based in Brazil. We identified complementary Brazilian journals with the Scimago Journal and Country Rank (SJR) 2020^7^ ^,^ ^8^. The SJR is a public access portal that includes scientific indicators from journals and countries, developed based on information contained in the Scopus database. This allows the comparison and analysis of journals according to major scientific themes and subject categories. Our search included journals under the subject area ‘’Medicine’’, subject category ‘’Surgery’’ and “Brazil” as the regional filter. We performed a systematic search in the identified journals, following the same previously described search strategy, to identify works published in Brazilian journals that are not indexed in the platforms used in our search strategy. Figure 1 describes the adopted search strategy.

### Collection, summarization, and results reporting

We collected a standard set of information from each of the included studies using a spreadsheet created for this review in Microsoft Excel (Microsoft, Santa Rosa, California, USA, 2016). We recorded the authorship, year of publication, type of study and journal of publication, and information regarding the limitations of the DATASUS platform pointed out by the studies.

We used Grounded Theory, reported by Strauss and Corbin^9^, to analyze the main limitations of using the platform cited by the included studies. This methodology is an inductive process in which the most prevalent themes described in the selected studies are identified and used to generate analytical domains, which are deduced in the initial phase of data collection. Thus, the limitations cited in each study were initially grouped into emerging domains and, subsequently, into categories and subcategories. As this is a non-linear analysis process, the articles were re-examined at the end of the process to confirm that saturation was achieved.

## RESULTS

### Descriptive analysis

After carrying out the search strategy, we identified 36 studies on PubMed, 38 on the Web of Science, 123 on Embase, and 45 studies on the Scopus platform. In parallel, we identified 11 Brazilian journals on topics related to SAO and other surgical specialties through the SJR. The search in these journals identified a total of 17 papers. Thus, we analyzed 248 works in the first stage of the search and, after reading the full texts, we included 47^5^ ^,^ ^10^ ^-^ ^55^ in the final analysis (Figure 1).

All works included in the search were original articles. Most (12.76%, n=6) were published in the Journal of The Brazilian College of Surgeons (RCBC) and the most common type of study found were retrospective studies (40.43%, n=19), followed by epidemiological studies (25.53%, n=12). The works included were published between 2009 and 2022 (Table 1).

**Table 1:** Summary of included works.

Authorship	Year	Study design	Journal
Truche et al.^5^	2020	Retrospective study	World Journal of Surgery
Do Nascimento et al.^10^	2022	Retrospective study	Brazilian Archives of Digestive Surgery
Ferreira et al.^11^	2018	Retrospective study	Brazilian Journal of Orthopedics
Tonatto-Filho et al.^12^	2019	Retrospective study	Brazilian Archives of Digestive Surgery
Olijnyk et al.^13^	2022	Retrospective study	Journal of the Brazilian College of Surgeons
Do Nascimento et al.^14^	2021	Retrospective study	Journal of the Brazilian College of Surgeons
De Magalhães et al.^15^	2017	Descriptive study	Brazilian Archives of Neurosurgery
De Magalhães, Mendes e Oliva^16^	2019	Descriptive study	Brazilian Archives of Neurosurgery
De Magalhães et al.^17^	2017	Descriptive study	Brazilian Archives of Neurosurgery
De Magalhães et al.^18^	2016	Descriptive study	Brazilian Archives of Neurosurgery
Dos Santos et al.^19^	2020	Retrospective study	Columna
Felício et al.^20^	2017	Descriptive study	Brazilian Archives of Digestive Surgery
Stolnicki e Teixeira^21^	2020	Cross-sectional study	Brazilian Journal of Orthopedics
Dos Santos, Cavasana e de Campos^22^	2017	Retrospective study	Journal of the Brazilian College of Surgeons
Malavolta et al.^23^	2017	Retrospective study	Brazilian Journal of Orthopedics
Garcia et al.^24^	2022	Descriptive study	Journal of the Brazilian College of Surgeons
Covre et al.^25^	2019	Epidemiological study	Journal of the Brazilian College of Surgeons
Oliveira et al.^26^	2022	Epidemiological study	Journal of the Brazilian College of Surgeons
Anacleto et al.^27^	2022	Epidemiological study	Brazilian Journal of Cardiovascular Surgery
Asano et al.^28^	2012	Epidemiological study	Obesity Surgery
Barros et al.^29^	2015	Epidemiological study	Revista Panamericana de Salud Publica
Bicudo et al.^30^	2021	Epidemiological study	International Journal of Clinical Practice
De Andrade et al.^31^	2022	Epidemiological study	Journal of Clinical Medicine
De Macêdo Filho et al.^32^	2021	Retrospective study	World Neurosurgery
Dos Santos et al.^33^	2017	Retrospective study	Journal of Global oncology
Everling et al.^34^	2020	Cross-sectional study	Gastroenterology Archives
Guimaraes et al.^35^	2022	Epidemiological study	Brazilian orthopedic record
Khalil et al.^36^	2021	Retrospective study	Journal of heart surgery
Kelles et al.^37^	2014	Retrospective study	Brazilian Archive of Digestive Surgery
Knobel et al.^38^	2020	Epidemiological study	Brazilian Journal of Gynecology and Obstetrics
Lemos et al.^39^	2018	Epidemiological study	Journal of the Brazilian Medical Association
Luciano et al.^40^	2018	Descriptive study	Medicine LWW
Magalhães et al.^41^	2022	Epidemiological study	Brazilian Vascular Journal
Nascimento et al.^42^	2016	Observational study	Annals of Vascular Surgery
Nascimento et al.^43^	2021	Epidemiological study	Cancer Epidemiology
Authorship	Year	Study design	Journal
Olijnyk et al.^44^	2014	Cross-sectional study	World Journal of Surgery
Trindade et al.^45^	2022	Observational study	The American Surgeon
De Souza et al.^46^	2018	Observational study	Hepatobiliary & Pancreatic Diseases International
Piegas et al.^47^	2009	Retrospective study	Brazilian Archives of Cardiology
Piegas et al.^48^	2011	Retrospective study	Brazilian Archives of Cardiology
Silveira et al.^49^	2022	Retrospective study	Frontiers in Endocrinology
Teivelis et al.^50^	2022	Descriptive study	Brazilian Vascular Journal
Wolosker et al.^51^	2021	Observational study	Annals of Vascular Surgery
Yu et al.^52^	2010	Retrospective study	Plos One
Korkes et al.^53^	2020	Descriptive study	Einstein (São Paulo)
Souza et al.^54^	2011	Retrospective study	Brazilian Vascular Journal
Faleiro et al.^55^	2022	Retrospective study	World Journal of Surgery

### Identified domains

Content analysis of the articles identified four predominant domains in the scientific literature regarding the limitations of using DATASUS in surgical research: (1) lack of data; (2) data reliability; (3) data accuracy; and (4) data integration. Table 2 shows the articles included in the analysis and the domains cited by each of them.

**Table 2:** Domains identified after the content analysis of each work through Grounded Theory. The presence of a dot (•) indicates that the domain was cited by the article.

First author, year	Lack of data	Data reliability	Data accuracy	Data integration
Truche et al, 2020^5^			•	
Do Nascimento, 2022^10^	•		•	
Ferreira, 2018^11^		•		
Tonatto-Filho, 2019^12^	•			
Olijnyk, 2022^13^	•	•		
Do Nascimento, 2021^14^	•			
De Magalhães, 2017^15^			•	
De Magalhães, 2019^16^			•	
De Magalhães, 2017^17^		•		
De Magalhães, 2016^18^			•	
Dos Santos, 2020^19^			•	
Felício, 2017^20^	•			
Stolnicki, 2020^21^	•		•	•
Dos Santos, 2017^22^	•			
Malavolta, 2017^23^	•			
Garcia, 2022^24^	•			
Covre, 2019^25^			•	
Oliveira, 2022^26^	•			
Anacleto et al, 2022^27^	•		•	
Asano et al, 2012^28^	•	•		
Barros et al, 2015^29^	•			
Bicudo et al, 2021^30^	•			
De Andrade et al, 2022^31^	•	•		
De Macêdo Filho et al, 2021^32^	•	•		
Dos Santos et al, 2017^33^		•		
First author, year	Lack of data	Data reliability	Data accuracy	Data integration
Everling et al, 2020^34^	•			
Guimarães et al, 2022^35^	•		•	
Khalil et al, 2021^36^	•			
Kelles et al, 2014^37^	•			
Knobel et al, 2020^38^	•			
Lemos et al, 2018^39^	•			
Luciano et al, 2018^40^	•			
Magalhães et al, 2022^41^	•	•		
Nascimento et al, 2016^42^	•	•	•	
Nascimento et al, 2021^43^	•			
Olijnyk et al, 2014^44^		•		
Trindade et al, 2022^45^	•	•		
De Souza et al, 2018^46^		•		
Piegas et al, 2009^47^	•			
Piegas et al, 2011^48^	•			
Silveira et al, 2022^49^	•			
Teivelis et al, 2022^50^	•			
Wolosker et al, 2021^51^	•			
Yu et al, 2010^52^		•		
Korkes et al, 2020^53^	•			
Souza et al, 2011^54^		•		
Faleiro et al, 2022^55^	•			
Total number of articles that referenced each domain	34	14	11	1

### Lack of data

DATASUS lack of data was the most prevalent domain among the four found (72.34%, n=34). In this domain, the authors stated that the absence of some clinical information that is not collected by the platform made it impossible to carry out an in-depth analysis, with less bias in their work. Among the missing information, individual information for each patient^38^ ^,^ ^39^ ^,^ ^43^ ^,^ ^48^ ^,^ ^49^ stands out, such as previous clinical comorbidities^9^ ^,^ ^12^ ^,^ ^13^ ^,^ ^21^ ^,^ ^23^ ^,^ ^28^ ^,^ ^32^ ^,^ ^37^ ^,^ ^42^ ^,^ ^47^, sex^13^ ^,^ ^21^ ^,^ ^36^, age^13^ ^,^ ^21^ ^,^ ^36^, nutritional status^9^ ^,^ ^13^, and socioeconomic parameters29,40. As for the surgical aspect, the authors highlighted that the lack of data about the surgical method used^12,13,25,30,51^ represented a bias in their analyses. Also, information about the postoperative period of patients, such as the need for reintervention, readmission, and the rate of postoperative complications are also not recorded on the platform^9^ ^,^ ^12^ ^,^ ^13^ ^,^ ^19^ ^,^ ^23^ ^,^ ^27^ ^,^ ^32^ ^,^ ^34^ ^,^ ^35^ ^,^ ^47^ ^,^ ^51^ ^,^ ^53^. Information on the average length of stay of the patient in each hospital is also not available^40^.

Authors also raised concerns related to the lack of data on specific procedures. Considering that two studies dealt with the surgical management of acute appendicitis, the authors emphasized that the lack of information on the degree of organ involvement negatively influenced their analyzes^13^ ^,^ ^21^. Tonatto-Filho et al.^11^ pointed out that the platform does not have numbers on the percentage of deaths among patients waiting in the queue for bariatric surgery in Brazil.

The platform also lacks data in specific surgical areas, such as Orthopedics, as highlighted by Malavolta et al.^22^.

Another theme identified in this domain is missing data, even in information that is collected by the platform. Malavolta et al.^22^ pointed out that, in certain years, some Brazilian states did not record rotator cuff repair surgeries on the DATASUS platform, which represents a potential limitation of its use. In the same vein, Garcia et al.^23^ reported that mortality related to unilateral inguinal hernia was not recorded on the platform by some regions during the period covered by their study.

Several authors also highlighted the lack of information about the private health system on the DATASUS platform^14^ ^,^ ^16^ ^,^ ^17^ ^,^ ^20^ ^-^ ^22^ ^,^ ^31^ ^,^ ^41^ ^,^ ^45^ ^,^ ^48^ ^-^ ^50^ ^,^ ^55^.

### Data reliability

The second domain identified concerns the reliability of data provided by DATASUS (29.78%, n=14). The main aspect highlighted by the authors is dependence of the platform on filling out of forms in the executing hospitals, which generates the possibility of bias in the reporting of information^32^ ^,^ ^33^ ^,^ ^41^ ^,^ ^44^ ^,^ ^45^ ^,^ ^54^, with a probability of including multiple or wrong diagnoses in the database^16^, in addition to the risk of underreporting^31^ ^,^ ^46^. Forms are usually filled by administrative technicians and non-medical professionals, which increases the risk of bias in the information provided^52^. Also, Nascimento et al.^42^ pointed out that as data entry is not systematically verified by a platform committee, random inaccuracies must be considered in the registration, especially for non-mandatory fields. DeCastro et al.^10^ emphasized that, due to inputting errors in the platform by the executing hospitals, its data are based on estimates and may interfere with the accuracy of study results.

Data reliability may also vary depending on the surgical volume of the hospital responsible for reporting on the platform. Asano et al.^28^ emphasized that, while hospitals with higher surgical volume generally have standardized procedures for reporting their data on the DATASUS platform, low-volume hospitals do not have such protocols and, therefore, coding errors and biases in the content reported on the platform may be present in these centers. Olijnyk et al.^12^ pointed out that the specification of the access route in cholecystectomies, for example, is information subject to such bias.

### Data accuracy

The third most prevalent domain identified in the literature concerns the accuracy of the data displayed on the platform (23.40%, n=11). The consulted literature highlighted that the registration of different surgically manageable conditions under the same International Classification of Diseases (ICD)^9^ ^,^ ^20^ or under the same code determined by the platform^14^ ^,^ ^15^ ^,^ ^17^ ^,^ ^18^ ^,^ ^27^ ^,^ ^35^ represented a potential bias in the analyzes due to the risk of data oversizing. Nascimento et al.^42^ pointed out that, although DATASUS has existed since 1992, the current coding system was implemented in 2008, and the effect of the learning curve can be considered in the first years.

For example, given that the single DATASUS code 04.03.02.013-1 covers both the microsurgical treatment of peripheral nerve tumors and neuromas, the results generated by the code include both surgeries performed for the treatment of peripheral nerve tumors and tumor lesions, which reduces the accuracy of the data displayed on the platform^15^. The same limitation was noted with use of the ICD, given that the ICD-10 K80 code contemplates a variety of diagnoses, such as cholelithiasis with acute or chronic cholecystitis (with or without obstruction), cholelithiasis without cholecystitis, gallstones with cholangitis (with and without obstruction), and gallbladder and biliary tract calculus with and without cholecystitis^9^.

### Data integration

The domain with the lowest prevalence concerns the integration of platform data (2.32%, n=1), since the DATASUS platform is responsible for aggregating information from different databases, such as the CNES, the SIA-SUS and SIH-SUS. For Stolnicki and Teixeira^20^, the system does not allow for integration of the hospitalization (SIH-SUS) and outpatient (SIA-SUS) databases.

## DISCUSSION

We conducted a scoping review of Brazilian and international journals to collect and summarize information from scientific articles in surgery and specialties involved with surgery, which used the DATASUS platform as a primary source of data. After the content analysis of these works, we verified that the currently available literature recognizes that the main limitations of this platform concern the lack of data, data reliability, data accuracy, and data integration.

Initially, we should note that hospital production that occurs with private resources or by health insurance plans is not part of this database, which aggregates information from philanthropic hospitals, public hospitals managed by SUS, and hospitals that receive patients from SUS^56^. However, although it has been cited by some authors, the non-inclusion of information on patients from the private system should not be considered the main limiting agent of the use of this platform for scientific research, given that 75% of the Brazilian population depends exclusively on SUS^60^.

The lack of data, especially in the SIH-SUS, was the main limitation of the platform mentioned by the included articles, considering that this was presented on two aspects: 1) information that is collected by DATASUS but is not properly made available, and 2) information that is not collected by DATASUS but whose collection would contribute to the construction of a solid information system. In the first category, the authors highlighted that some variables made available by the platform had several missing values, that is, “blank cells”. In the second category, a considerable obstacle in observational studies using the system is the missing information on patients, such as relevant epidemiological descriptions and comorbidities and secondary diagnoses. The lack of data in both mentioned categories constitutes an important limitation of the platform because, as noted, it causes underutilization of the DATASUS platform in surgical epidemiological studies. For example, of the eleven Brazilian journals selected for our study, only five published papers that used the DATASUS platform as a primary data source.

[Fig ch1]

**Flowchart 1. ch1:**
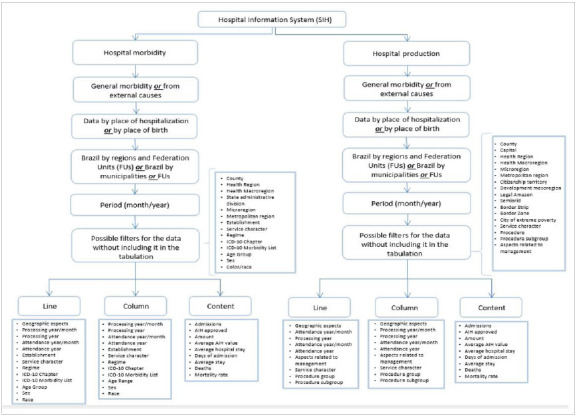

The way in which the platform receives coding inputs of procedures and their morbidities raises concerns about the accuracy of the data presented, an aspect commented on by 41.7% of the studies included in this work. Diagnosis by ICD, as noted on the platform, extends only to the category level, not exploring subcategories, which makes it impossible to delve deeper into diagnostic peculiarities^9^ ^,^ ^20^. As for hospital procedures, these are codified by the SUS Procedures, Medications, and OPM Table Management System (SIGTAP/SUS) and can be ambiguous when dealing with procedures used in the surgical management of more than one pathology because, technically, the remuneration for a procedure does not change according to its application^5^ ^,^ ^14^ ^,^ ^15^ ^,^ ^17^ ^,^ ^18^.

Another aspect to be highlighted it the reliability of the data presented on the platform, a concern that is due to the dependence of DATASUS on filling of forms by the executing hospitals themselves. As highlighted by Barbosa (2019)^1^, the collection of information on the production in health facilities involves manual recording on printed forms, which are then reinserted into virtual information systems, implying rework that often leads to integrity losses in the process of gathering information. In addition, works in the literature warn about the possible manipulation of these codes for financial favoring of health institutions^58^.

Another limitation regarding the reliability of the data provided by the SIH-SUS is that the unit of the system is the hospitalization, represented by the AIH, and not the individual^1^. Thus, each patient’s contact with hospital care generates a new record, so that a single hospitalization can cause the issuance of several AIHs for the same patient^58^, which generates overestimation of the data made available based on approved AIHs. Also, attention should be paid to the registration of erroneous diagnoses through the AIH. A study carried out to identify the validity of the information available in the SIH-SUS in a hospital in the Federal District found that 91% of the diagnoses indicated in the AIHs did not correspond to the diagnoses described in the medical records^59^.

The lack of integration between the data of the different information systems that make up the DATASUS platform, although being the least mentioned domain in this scope review, also requires careful analysis. Previous works reported that the lack of payment reflects the need for individual information in each technical area, thus justifying the constitution of a new information system for each new information need^60^. Consequently, many systems were created that still do not integrate and often duplicate information, contributing to results overestimation.

Despite the DATASUS limitations elucidated by the study, the positive impact of the implementation of the DATASUS platform with the Information Systems must be highlighted. Objections to retrospective, cross-sectional studies that establish correlations relevant to the formulation of public policies and hypotheses must consider that the continuous use of the data source allows for greater understanding of it and improvement in the development of methodologies in health indicators^58^. The use of more than one Information System can be a way to circumvent imposed biases and strengthen studies focused on the surgical field.

Finally, the results of this scope review highlight the lack of scientific literature that specifically seeks to explain the origin of the limitations of the DATASUS platform and how to mitigate them. The main limitations of this scope review are the non-inclusion of articles published in other scientific journals that may not have been identified through the SJR and the non-inclusion of scientific articles that, despite having used the DATASUS platform as a primary source of data, could not be identified based on the descriptors used in our search. Despite these limitations, for the first time, we were able to conduct a rich analysis of the main limitations of using this platform in studies on surgery.

## CONCLUSION

Although the DATASUS platform is the largest source of data and information on surgical procedures in SUS, the available scientific literature on its main limitations remains scarce. The works currently available recognize that the main limitations of this platform pertain to lack of data, data reliability, data accuracy, and data integration.

The objective of this scope review was the synthesis of the current scientific literature about the main limitations of this platform, to serve as a basis for future public policies that seek to strengthen it. It was possible to perceive that the lack of supply of the system with specific information about the procedures and individual aspects of the patients represented an important barrier for the production of reliable data. Furthermore, we highlight the divergences caused using AIHs as a unit in the SIH-SUS, which, added to the lack of data integration from this system with the SIA-SUS and SIH-SUS, ends up reducing the reliability of the data generated by DATASUS. Given this scenario, actions are needed to propose solutions to the DATASUS limitations presented in this work, such as carrying out studies to measure the quality of data provided on the platform and educating physicians and managers for more accurate and adequate filling of the forms. 
